# Wideband Microwave Photonic Circulator Using Two Asymmetric Partial-Height Triangle Ferrites

**DOI:** 10.3390/ma15196689

**Published:** 2022-09-27

**Authors:** Yong Wang, Biaogang Xu, Wenlong He, Hou Ian

**Affiliations:** 1 Institute of Applied Physics and Materials Engineering, University of Macau, Macau 999078, China; 2 College of Electronics and Information Technology, Shenzhen University, Shenzhen 518060, China

**Keywords:** circulators, photonic crystal, microwave photonic, ferrites, 5G, wideband

## Abstract

Broadband 5G communication requires the operation of nonreciprocal devices in the Ku band. A wideband photonic crystal circulator is implemented by introducing two partial-height triangular Ni-Zn ferrites into the Al_2_O_3_ ceramic rod-arrays. The asymmetric sizes of the two equilateral triangles paired with self-matching effectively extend the bandwidth of the circulator eight times over that of the symmetric scheme. Numerical simulations demonstrate that the photonic crystal circulator can obtain a bandwidth of 1.00 GHz with an isolation 25.75 dB and an insertion loss 0.381 dB through optimized matched triangle size ratio, suitable for applications in future communication systems.

## 1. Introduction

**Figure 1:**
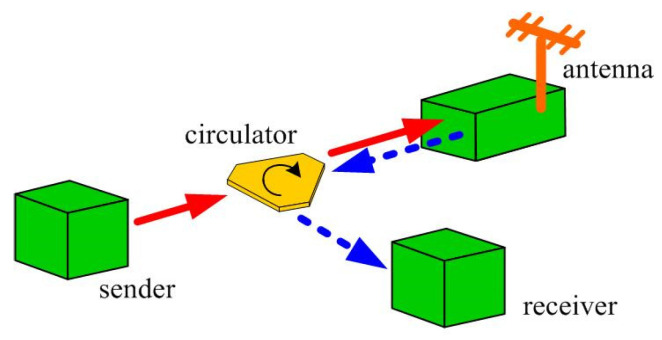
Circulator in the communication systems.

Nonreciprocal passive circulators play an important role in telecommunication, serving functions such as directional control, duplexing, and isolation of incoming and outgoing signals. For example, they have been applied to achieve a fully separation between upward- and downward-transmitting signals in communication systems [1,2,3], such as the one shown in Figure 1. With the current development of 5G communication technologies for ever denser congregations of signals, communicating bands are moving towards the higher frequency end, where the millimeter wavelength Ku band around 26 GHz is one of them. Circulators operating in this band are therefore needed to serve similar functions described above in order that the next generation communication system can be realized.

Since the photonic crystal (PhC) was first investigated [4,5], microwave photonic devices based on PhCs for high-frequency operations have drawn considerable concern and attracted much research [6,7,8,9,10,11,12]. PhCs have been shown to be an effective medium for transmitting as well as controlling electromagnetic waves, which can readily facilitate the miniaturization and integration of devices at the same time [13,14,15,16,17,18].

In recent years, photonic crystal circulators [19,20,21,22] have attached much attention because of their design flexibility and potential applications in optical electronic integrated circuits. A windmill-format PhC circulator was designed by using air-hole photonic crystal, which has a relative bandwidth of 0.045% [19], and a W-shaped circulator with relative bandwidth of 0.052% was envisaged by using a type of carefully designed PhC cavity at 1550 nm [20]. Additionally, a dielectric-rod-array-based terahertz PhC circulator obtained a relative bandwidth of 2.7% in their numerical simulation [21]. Based on a square-lattice dielectric-rod array, a T-shaped microwave photonic circulator was investigated at X-band by using some coupled magneto-optical rods and a side-coupled cavity [22]. These diversified schemes suggest that photonic crystals increase flexibility for implementing different kinds of circulators that meet the differing requirements of future optoelectronic integrated modules. Nonetheless, these same schemes are limited in bandwidth due to the limited resonance linewidth of the cavities used, poor impedance matching, and other reasons, restricting their applicability to future communication appliances.

To broaden the usable bandwidth of a PhC circulator, here we present a novel wideband microwave photonic circulator scheme that makes use of self-matching technique (SMT) on triangular shaped ferrites of partial heights. That is, their heights do not extend to the full height of the PhC rods. Unlike the external matching broadening method (EMBM) [23], SMT relies on the adjustment of the distinct radii of the circles circumscribing the upper and the lower triangular ferrites to achieve an ideal impedance match. The bandwidth of the designed circulator is improved substantially over previous designs based on spherical ferrites [18], without the augmentation of any other metal plates. The ferrite materials have high resistivity but low eddy current loss, causing them to possess a high dielectric constant at relatively high frequency bands [24]. This fact translates into a macroscopically large refraction index, allowing the ferrites in the circulator to have a steering ability for incident microwaves. In the case studied here, when the radii of the circles are 3.25 mm and 3.45 mm, the bandwidth of the circulator is substantially extended to 1 GHz, corresponding to a relative bandwidth of 3.8%, while the peak isolation and insertion loss reach 25.75 dB and 0.381 dB, respectively. Therefore, the wideband implementation provides a feasible scheme for realizing a high-performance microwave photonic circulator for future 5G communication systems.

## 2. Design of the Photonic Crystal Circulator

**Figure 2:**
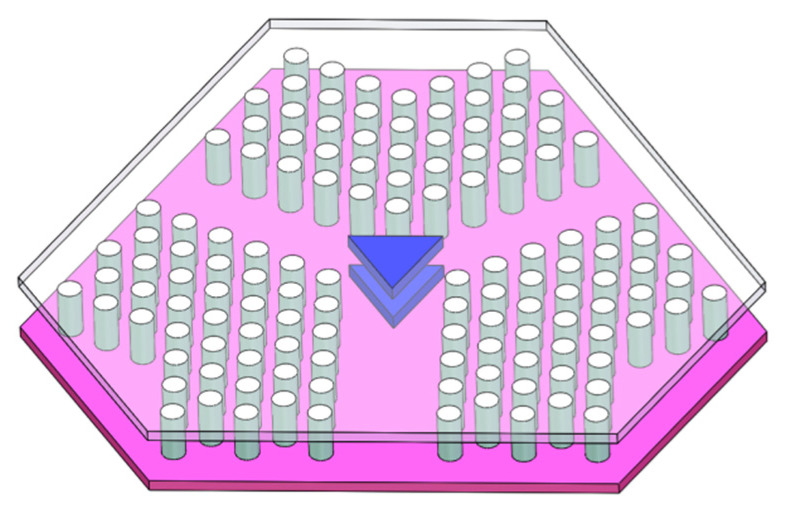
Design of the Y-typed microwave photonic circulator with two triangular ferrites.

In Figure 2, the design drawing of the microwave photonic circulator is formed by a Y-type photonic crystal waveguide (PCW) and two central ferrites (blue). The PCW consists of three 2-D triangular lattice PCs (white), which are precisely immobilized by the basal plate (pink). The PCW acting as the rectangular waveguides has width w of 8.64 mm and height h of 4.32 mm, following the standard size of a WR34 waveguide, along which TE_10_ mode is the only transmitting mode. Two triangular Ni-Zn ferrites (blue) are placed at the center of the waveguides, where the radius of the circle circumscribing the ferrites is 3.35 mm to match the waveguide width.

**Figure 3:**
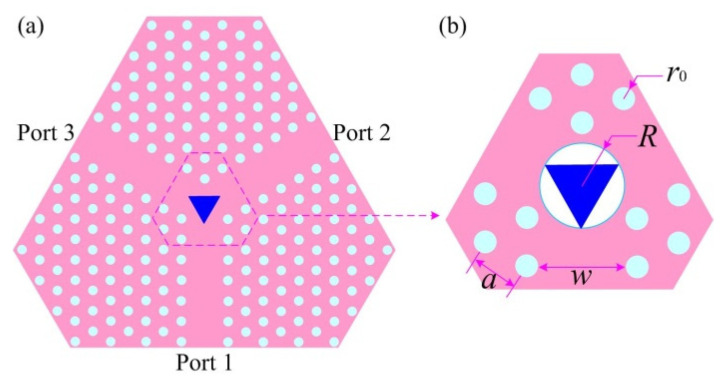
(**a**) The section view of the Y-typed PCW and ferrites; (**b**) the parameter annotations of the PhC and ferrites.

Figure 3a shows the sectional view of the microwave photonic circulator. In Figure 3b, the dielectric rods (Al_2_O_3_) with a relative permittivity of 9.2 are 4.32 mm in height and spaced apart with a lattice constant *a* of 3.5 mm. The radius of the circumscribing circle is denoted by R and the rod radius is denoted by *r*_0_. The TE_10_ mode under interest is associated with a lenient photonic bandgap (PBG) that permits electromagnetic waves only within a certain frequency range to transmit in the waveguides. This PBG can be tuned by adjusting the ratio of *r*_0_ to the constant *a*.

## 3. Analytical and Numerical Results

**Figure 4:**
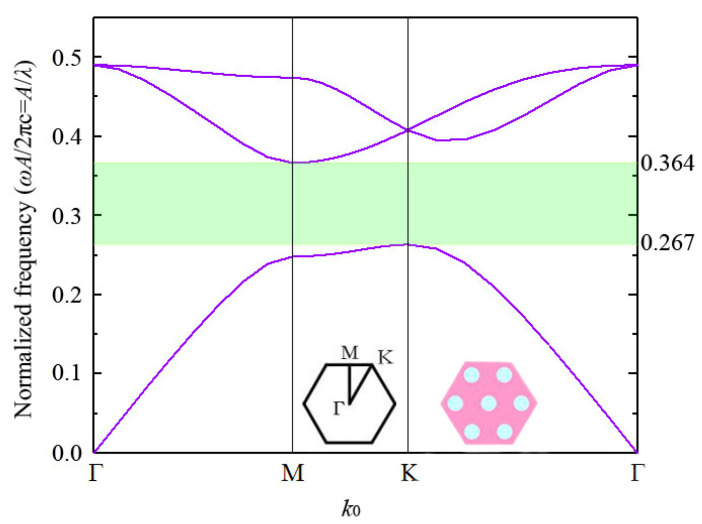
The PBG of the PhC with *r*_0_ = 0.19*a*.

In Figure 4, the light green area shows the PBG of the PhC. When *r*_0_ *=* 0.19*a*, the normalized frequency (2πc/*a*) obtains the broadest optimal range, from 0.267 to 0.364, corresponding to a frequency range of 22.89 to 31.20 GHz.

In the millimeter waveband [25], the susceptibility to be computed depends on the permeability tensor $\left[\mu_{r}\right]$, which can be expressed as

(1) $$\left[\mu_{r}\right]=\mu_{0}\left[\begin{array}{ccc} \mu & j\kappa & 0\\ -j\kappa & \mu & 0\\ 0 & 0 & 1 \end{array}\right],$$

where the diagonal element

(2) $$\mu =1+\omega_{0}\omega_{m}/\left(\omega_{0}^{2}-\omega^{2}\right),$$

the non-diagonal element

(3) $$\kappa =\omega_{0}\omega_{m}/\left(\omega_{0}^{2}-\omega^{2}\right),$$

where $\omega_{0}=\mu_{0}\gamma H_{0}$ and $\omega_{m}=\mu_{0}\gamma M_{s}$. In addition, $H_{0}$ is external dc magnetic field, while the gyromagnetic ratio $\gamma =1.759\times 10^{11\;}C/\mathrm{kg}$ and the saturation magnetization $M_{s}=2.39\times 10^{5}\;A/m$.

**Figure 5:**
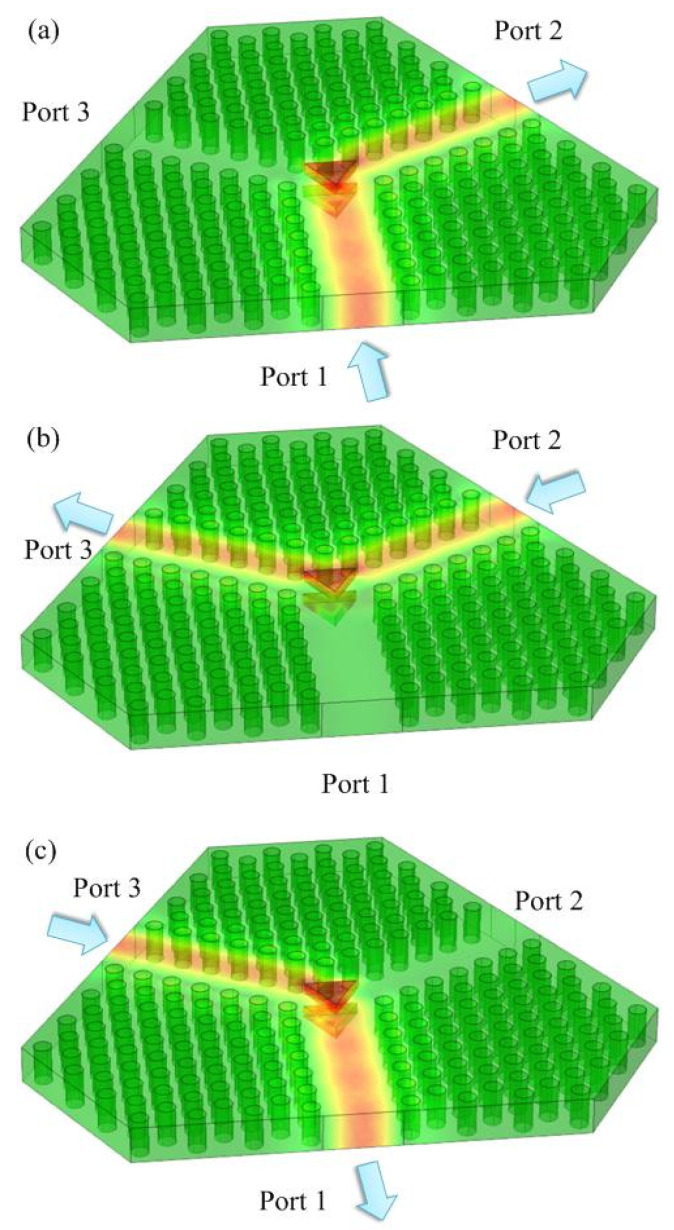
(**a**) The power distribution in the PhC circulator at 26.35 GHz when signal incident from Port 1; (**b**) signal incident from Port 2; (**c**) signal incident from Port 3.

The external characteristics and function of the Y-typed PhC circulator are simulated using finite element method according to the following equation:

(4) $$\varepsilon^{-1}\cdot \nabla \times \left({\left[\mu_{r}\right]}^{-1}\cdot \nabla \times \vec{E}\right)=\omega^{2}/c^{2}\cdot \vec{E},$$

where the relative dielectric constant ε=13.5 for the Ni-Zn ferrite, while $\left[\mu_{r}\right]$ is described by Equation (1), and $\vec{E}$ is the electric field intensity. In addition, ω is the circular frequency of the incident electromagnetic wave. The parameters we use as a baseline reference are *a* = 3.5 mm, *r*_0_ = 0.19*a*, *R* = 3.35 mm, $H_{0}$ = 0.038 T. With these numbers, the power distribution of the electromagnetic field is simulated using COMSOL using the high-frequency package, where the field powers are shown as color heat maps layered over the CAD renderings of the circulator in Figure 5. Setting the central frequency to 26.35 GHz, the signal incident from Port 1 is simulated in Figure 5a. The signal receives a 120° rotation at the two triangular ferrites area in the propagating path of Port 1 to Port 2, and there is almost no power going to Port 3. Similarly, when signal is incident from Port 2 or Port 3, the corresponding power distributions in the circulator are shown in Figure 5b,c, respectively. The function of the Y-typed PhC circulator is displayed by the numerical simulations.

**Figure 6:**
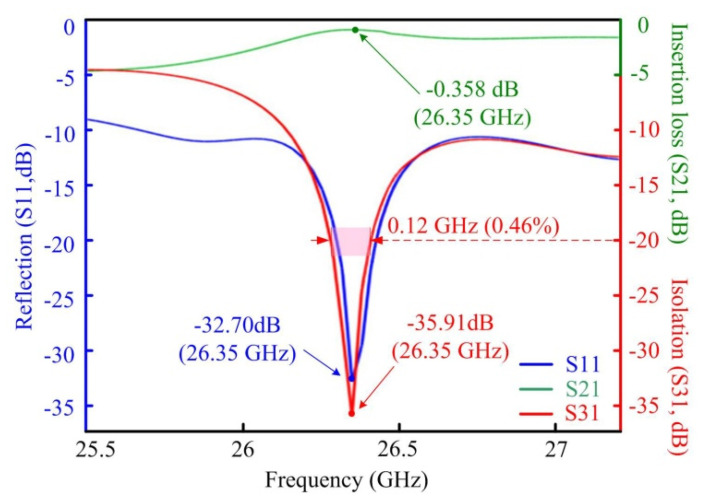
External characteristics of the microwave photonic circulator. The blue S11 reflection dip coincides with the red isolation S31 dip, showing a suitable working bandwidth (indicated by the pink band) for the designed circulator. In this band, incident signals into port 1 would be optimally directed to port 2 without being either reflected back into port 1 or directed towards port 3.

**Figure 7:**
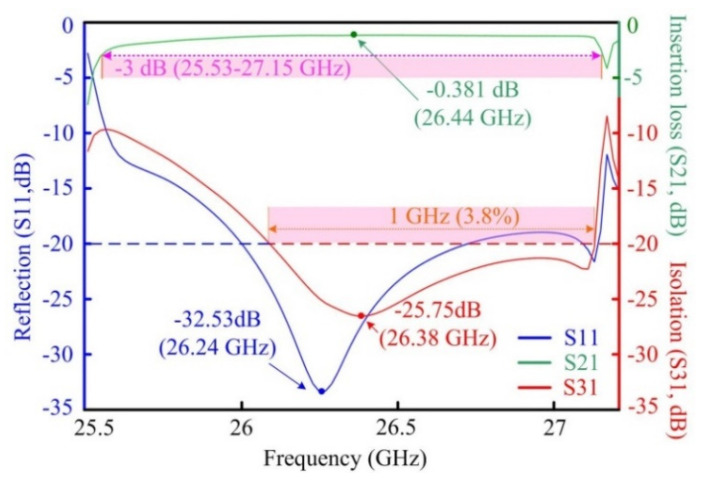
External characteristics of the modified PhC circulator.

It can be observed that the periodically spaced PhC rods guides the incident waves well and confined them in the void passage because of the good internal reflections of the crystalline material, no matter the incidence port. In addition, the Ni-Zn triangular ferrites demonstrate strong refraction for microwave signals within the Ku-band. Directional steering is optimized repeatedly over the sizing of the ferrites in the simulation to ensure best refraction at 120 degrees. Combining these two facts, the leakage is limited, as verified in the simulation results of Figure 6 and Figure 7 below for simulation in the frequency domain for the incident fields into port 1 given in Figure 5.

In microwave engineering, the external characteristics of passive devices are usually obtained by the parameters of S_21_ and S_31_, respectively. Similarly, the performance parameters of insertion loss *τ* and isolation *α* for our designed circulator should be expressed as follows:

*τ* = −S_21_ = 10log(P_21_/P_1_) (5)

and

*α* = −S_31_ = 10log(P_31_/P_1_), (6)

where P_1_ represents the input power, and P_21_ and P_31_ express the output power of the other two ports. We also study the reflection of the input Port 1 for our designed circulator, which can be represented as:

S_1__1_ = 10log(P_R1_/P_1_), (7)

in which P_R1_ represents the reflection power from Port 1.

When signal is incident from Port 1, the curves of isolation, insertion loss, and reflection of the microwave photonic circulator are simulated with the frequency range of 25.5 to 27.2 GHz in Figure 6. The lowest reflection and insertion loss of the circulator are obtained as −32.70 dB and 0.358 dB, while the peak isolation reaches 35.91 dB at 26.35 GHz. However, the bandwidth is about 0.12 GHz, in which the isolation keeps above 20 dB. In the current design with two symmetric triangular ferrites (*R* = 3.35 mm mentioned above), the circulator only has a narrow relative bandwidth of 0.46%.

Aiming to broaden the operating bandwidth of this microwave photonic circulator, we apply the SMT, which is easier and more feasible than the EMBM [23]. Circulators achieve impedance matching through the EMBM by placing aluminum matching gaskets with the same shape symmetrically outside the central ferrites. In the circulator, the gaskets act as a matcher and achieve impedance matching between all ports and the central junction as much as possible. More conveniently, without adding any other metal gaskets in our modified plan, next, we only need to adjust the sizes of the two triangular ferrites respectively and achieve a perfect match with appropriate sizes. The optimal bandwidth is obtained by repeatedly simulating for different combinations of sizes of ferrites, rods, and rod spacings. During the simulation optimization, the directional pointing of the ferrite vertices with respective to the direction of the incident wave is also important.

When the radius of the circle circumscribing the upper ferrite is reduced to be 3.25 mm and that of the lower ferrite is enlarged to be 3.45 mm, there is a good matching between the ports and the asymmetric ferrites (the two ferrites maintain a thickness of 1.35 mm). In the modified plan, the bandwidth is substantially extended to be about 1 GHz, as shown in Figure 7, in which the isolation of the circulator keeps above 20 dB. The optimal insertion loss *τ* reaches 0.381 dB at 26.44 GHz and the peak isolation *α* is 25.75 dB at 26.38 GHz. The input port gets the lowest reflection of −32.53 dB at 26.24 GHz. It is obvious that the relative bandwidth of the modified design is effectively extended to 3.8% by using SMT in the numerical simulation, which is an improvement of eight times compared to that of the symmetric scheme.

## 4. Conclusions

We employed SMT bandwidth broadening technology to introduce a novel PhC circulator using two triangular Ni-Zn ferrites in a 2-D triangular PhC lattice. Under optimized parameter settings, the effective bandwidth can reach 1 GHz about the center frequency of 26.38 GHz, with an isolation of 25.75 dB. Therefore, we proved that the self-matching technique can be realized by the asymmetry of the sizes of two triangular ferrites. Installed at the center of the circulator, these ferrites eliminate the reliance on external devices, making the circulator more compact and reliable for long-term usage. In particular, the central ferrites have a circumscribing radius about 3 mm, while the guiding PhC rods are about 0.7 mm in radius. These parameters lie well within the technical limits of conventional micromachining fabrication. The upper and lower plates of the circulator are made of common metallic materials, without any special requirements. Therefore, the design is good for real-world operation of future 5G communication hardware.

In this work, we focus only on isolation aspect of the ferrites with respect to microwaves and their triangular shapes are considered equilateral and aligned. The potential applications of triangular ferrites for other microwave signal processing remain to be explored in future works.
